# Structural and functional network characteristics and facility delivery among women in rural Ghana

**DOI:** 10.1186/s12884-017-1611-2

**Published:** 2017-12-19

**Authors:** Leslie E. Cofie, Clare Barrington, Kavita Singh, Sodzi Sodzi-Tettey, Susan Ennett, Suzanne Maman

**Affiliations:** 1 Center for Interdisciplinary Research in Women’s Health, University of Texas Medical Branch, 301 University Blvd, Galveston, TX 77555-0128 USA; 2 Department of Health Behavior, University of North Carolina, Gillings School of Global Public Health, 302 Rosenau Hall, CB #7440, Chapel Hill, NC 27599-7440 USA; 3 Carolina Population Center, University of North Carolina, CB#81200, Chapel Hill, NC 27599-7440 USA; 4 Department of Maternal and Child Health, University of North Carolina, Gillings School of Global Public Health, 401 Rosenau Hall, CB #7445, Chapel Hill, NC 27599-7445 USA; 5 Institute for Healthcare Improvement, Accra, Ghana

**Keywords:** Social networks, Maternal health, Health facility delivery, Homebirth, Ghana

## Abstract

**Background:**

Health facility births contribute to the prevention of maternal deaths. Although theoretical and empirical evidence suggest that social network characteristics influence facility delivery, examination of this relationship in sub-Saharan Africa is limited. We determined whether network structural and functional characteristics were associated with, or had an interactive effect on health facility delivery in rural Ghana.

**Methods:**

Data on mothers (*n* = 783) aged 15–49 years came from a Maternal and Newborn Health Referral (MNHR) project in Ghana, and included egocentric network data on women’s social network characteristics. Using multivariate logistic regression we examined the relationship between facility delivery and women’s network structure and functions, as well as the interaction between network characteristics and facility delivery.

**Results:**

Higher levels of instrumental support (e.g. help with daily chores or seeking health care [OR: 1.60, CI: 1.10–2.34]) and informational support (OR: 1.66, CI: 1.08–2.54) were significantly associated with higher odds of facility delivery. Social norms, such as knowing more women who had received pregnancy-related care in a facility, were significantly associated with higher odds of facility delivery (OR: 2.20, CI: 1.21–4.00). The number of network members that respondents lived nearby moderated the positive relationship between informational support and facility delivery. Additionally, informational support moderated the positive relationship between facility delivery and the number of women the respondents knew who had utilized a facility for pregnancy-related care.

**Conclusions:**

Social support from network members was critical to facilitating health facility delivery, and support was further enhanced by women’s network structure and norms favoring facility delivery. Maternal health interventions to increase facility delivery uptake should target women’s social networks.

## Background

In 2015, 66% of the world’s maternal deaths occurred in the sub-Saharan African region, which is also the region with the highest maternal mortality ratio (MMR) at 546 maternal deaths per 100,000 live births [1]. Most maternal deaths occur as a result of health risks associated with pregnancy, including hypertensive disorders of pregnancy, postpartum hemorrhaging, sepsis, complications from childbirth or unsafe abortions, malaria and anemia [2]. Many maternal deaths are preventable through health facility delivery with the assistance of a skilled birth attendant [3]. Facility delivery is considered the most efficient and cost-effective means of preventing maternal deaths, but in Africa over half of all births occur outside of health facilities [4, 5]. The MMR in Ghana is among the highest globally (32nd) with 319 deaths per 100,000 live births [1, 6]. According to the 2014 Ghana Demographic Health Survey (GDHS), 73% of births in the country occurred in health facilities. While 90% of births among urban populations in Ghana occurred in facilities, only 59% of births among rural populations were in facilities [7]. Over half of the population in Ghana lives in rural settings, which highlights the need to improve use of facility delivery in these regions.

Determinants of health facility delivery in sub-Saharan African countries like Ghana include distance to a facility, available transportation, affordable cost of facility care, maternal socio-demographics (e.g. age, parity, education level, wealth, health decision making autonomy), perceived needs/benefits of facility birth, and facility factors (e.g. health provider attitude, privacy, and quality of care) [8–10]. Researchers have argued, however, that studies on determinants of facility delivery are limited in part because most studies do not account for key social determinants, including social networks [4, 9]. An in-depth examination of the link between women’s network characteristics and their pregnancy related experiences could contribute to knowledge on improving uptake in facility delivery care. To address this gap in the literature, we examined the role of social networks in women’s use of health facility for delivery in Ghana.

A social network is a web of social relationships among a group of individuals that has both structural and functional characteristics (see Table 1) [11, 12]. Public health researchers are interested in how the structural and functional characteristics of social networks promote and influence health behaviors and ultimately health outcomes [13]. Network structural characteristics include network size, network members’ connectedness (density), demographic similarities (homogeneity) and emotional closeness of network members to each other (strength of tie) [14, 15].

**Table 1:** Definition of social network terms

Social network terms	Definitions
*Network structure*	Properties of the relationship among members within a network
Network Size	Number of members in a network
Frequency	How often an individual comes in contact with his/her network members
Proximity	How close network members live to a focal person
*Network function*	Resources exchanged among individuals in a network, such as social support and social influence
Social support	Different kinds of support provided and received by members within a network
Instrumental support	Aid/assistance provided by network members
Emotional support	Empathy, care, and understanding provided by network members
Information	Advice/ suggestions received from network members
Social influence	Shared norms and behaviors that impact one’s attitudes and behaviors
Injunctive norms	Perceptions of acceptable behaviors by network members. Members tend to be influenced by and adhere to such behaviors in order to avoid social sanctioning
Descriptive norms	Perceptions of common behaviors among network members. Individuals tend to adopt behaviors they believe to be normative among their network members.

Functional characteristics are the resources exchanged among individuals in a network such as social support and social influence in the form of social norms. Social support by network members can include: informational support (advice/suggestions), instrumental support (aid or assistance) and emotional support (empathy, care and trust) [16]. Social norms potentially impact one’s attitudes and behaviors and are conceptualized as both descriptive and injunctive [16–18]. Descriptive norms refer to the perceptions of behaviors that are common among network members. Individuals within a network tend to adopt the behaviors they believe to be normative among other network members [17]. Injunctive norms are perceptions of behaviors that are considered acceptable by network members, and individuals may feel influenced to adhere to those behaviors in order to avoid social sanctions [18].

Conceptually, social ties among network members are considered the structural basis on which social support and social norms impact health behavior [16, 19]. Not only do they have a direct effect on health, but they can also potentially operate by interacting to form underlying mechanisms that influence proximate health, including negative health or health promoting behaviors. In this study, we examine the association between network structural and functional characteristics and health facility delivery.

There is evidence of a positive association between network structure (e.g. network size, density, homogeneity, proximity of network members to one another, and frequency of contact) and health services use, including maternal care [20–24]. Research focusing on the relationship between network structure and facility delivery is limited. Edmond et al. (2012) found that network density, homogeneity and strength of ties among women in rural Bangladesh were not significantly related to facility birth [25]. As a result, they suggested a need to examine other structural network measures that may be associated with facility delivery.

In terms of functional network characteristics, first, a limited number of studies on the association between social support and facility delivery suggest that women who received informational support (advice to utilize facility-based delivery) and instrumental support (help with house chores and farming) during pregnancy were more likely to utilize facility delivery compared with women who did not receive these kind on support [25–27]. Second, community level social norms about the importance of facility birth have been positively related to women’s facility delivery [28]. Speizer and colleagues (2014) recently found that Ghanaian women who perceived that a higher proportion of husbands or mothers-in-law supported facility delivery, and those who perceived that a higher number of women in their community had facility birth, were significantly more likely to use facility delivery as well [29]. Research on network functions, which specifically focus on how social norms within women’s networks are related to their use of facility delivery, is limited.

Social network theories suggest that the interaction of network characteristics may be associated with facility delivery [16, 30, 31]. A qualitative study in rural Bangladesh examined husbands’ perceptions of social norms regarding facility delivery, and their provision of emotional, instrumental and informational support for women’s childbirth [32]. Husbands whose wives had facility births believed health facility delivery was necessary and provided their wives with different types of support during delivery, whereas husbands whose wives had homebirths believed that childbirth should be at home and were unsupportive of women’s facility delivery. The only study to date that examined the interaction of network structure (density, homogeneity and strength of ties) and network function (measured as perceived advice to deliver at a facility or home) was not significantly associated with facility delivery [25]. The authors acknowledged that the measures used in their study were likely unrepresentative of structural network characteristics that may potentially be linked with facility delivery.

Presently, no known study has examined the relationship between network structure and health facility delivery in sub-Saharan Africa and only one study, in rural Kenya, has quantitatively examined the relationship between social support and health facility birth [27]. Studies on social norms and facility delivery have mainly focused on community level norms [28, 29]. Future interventions to improve facility delivery access and use would greatly benefit from research examining which network structural and functional properties are associated with facility delivery among women. Additionally, examining whether the interaction of network structure and functions are associated with facility delivery can improve knowledge on the process by which social network is related facility delivery. To that end, the aim of our study is to determine whether characteristics of network structure and function (social support and social norms) are related to facility birth among rural Ghanaian women.

### Conceptual framework

While network members can influence health behavior through the provision of instrumental, emotional and informational support, network structural characteristics can potentially modify the relationship between social support and facility delivery by either augmenting or diminishing the types of support women receive. For example, among women who receive social support for facility delivery, those who live near large numbers of network members compared with those who do not, would be more likely to have facility delivery. This is because they would have many network members to depend on for support.

**Fig. 1:**
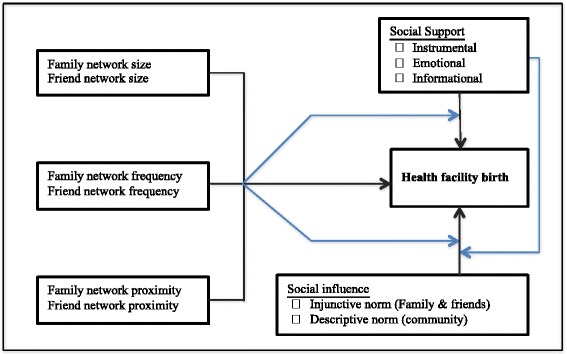
Conceptual model of the relationship between network characteristics and facility birth

As indicated in previous studies, constructs of social network theories are valuable tools for examining the relationship between network characteristics and health facility delivery. Network members play important roles in influencing decision-making regarding women’s pregnancy related care, and thus social networks matter in the decision to deliver at a health facility [9]. Figure 1 summarizes the relationship between social network structure, social support, social norms, and facility delivery. We hypothesize that network structure (larger size, higher frequency of contact with and closer proximity to network members) will be positively associated with facility delivery (Fig. 1). Also, informational, emotional and instrumental support from network members during last pregnancy and social norms favoring facility delivery will be positively associated with use of health facility birth. Network structural characteristics will moderate the relationship between social support, social norms, and facility delivery. Additionally, we hypothesize that social support will moderate the association between social norms and facility delivery.

## Methods

### Study setting

We used cross-sectional data from the endline evaluation of a Maternal and Newborn Health referral (MNHR) study, under Project Fives Alive (PFA) in Ghana [33]. The study sought to contribute to the overall PFA goal of reducing maternal and neonatal mortality by improving the referral process for pregnant women and newborns in need of comprehensive medical care. A household survey instrument was administered to women between January and March 2015 with the goal of assessing knowledge, practices and attitudes about maternal and newborn referrals. For the purpose of this study additional items were included to assess women’s perspectives on their social network characteristics during their most recent pregnancy. This study was approved by the Ghana Health Service Ethical Review Committee and exempted from ethics review by UNC-Chapel Hill’s Internal Review Board, as it was considered a program evaluation.

### Study design and sample

This study was conducted in three districts each in the Northern and Central regions of Ghana. Two of the districts in each region were designated as the intervention districts. The third district in each region was designated as the comparison district. The survey employed a 30 by N cluster sample design whereby 30 clusters of communities were randomly selected in the three designated districts in the Northern region (15 intervention communities and 15 comparison communities), and similarly 30 communities were selected in the Central region. The cluster sampling design is an efficient method of obtaining a representative sample in communities where collecting household census would be difficult [34]. The clusters were selected from a list of all communities in the districts of interest. From a comprehensive list of recently pregnant women in each community, seven women with recent births in the past 12 months were randomly selected in each cluster to be interviewed. This list was compiled through interviews with community health workers, traditional birth attendants and community leaders. Also, two of each pregnant woman’s closest neighbors were interviewed (age 15–49 years) because knowledge, attitudes and practices of women of all ages regardless of whether or not they had a delivery were considered valuable in informing improvement in health services use.

Data from the intervention and control communities in both regions were combined because our analysis is not significantly affected by the intervention implemented in the MNHR study. The sample in each region was 630 women (210 women with a recent birth in the past 12 months and 420 additional women of reproductive age), and a total of 1260 women were interviewed. We selected only women who had given birth in the past 3 years at the time of the survey in order to capture as large a sample of women as possible, while being mindful of concerns about recall bias (*n* = 818). We excluded women who had missing information on any key variables of interest resulting in an analytic sample of 783.

### Variables

We defined the outcome variable, health facility birth, as having delivered at a health facility during participant’s most recent pregnancy in the last 3 years.

Key independent variables included social network structural and functional characteristics. We adopted social network questions from existing measures and review of the network literature [17, 35–41]. We modified and tested the measures in the Northern and Central regions, in order to ensure that the questions were culturally relevant and reflect the local context of women’s experiences. Network structure variables included network size, frequency of interaction and proximity of network members to women during their last pregnancy. We created a continuous variable for network size by adding the responses of two items: “how many of your (1) relatives, and (2) friends, with whom you feel close to and can call on for help did you have any form of contact with during your last pregnancy?” We created a continuous variable for frequency of interaction with network members by summing two items: “How many of these (1) relatives, and (2) friends, did you see or interact with at least a few times a week during your last pregnancy?” Also, we created a continuous variable for proximity by summing two items: “How many of these (1) relatives, and (2) friends, lived in your village (community) during your last pregnancy?”

Network functional characteristics included social support and social norms. For social norms, we assessed injunctive norms using two variables: “how much do your (1) close relatives, and (2) friends, you described in the previous section approve of or encourage the use of health facilities for care during pregnancy and childbirth?” The response options were: strongly approve, approve, somewhat approve, do not approve, and not applicable. We recoded the response options into either higher approval (strongly approve or approve) or lower approval (somewhat approve, do not approve, or not applicable). For the descriptive norm variable participants were asked: “How many of the women you know of (e.g. relatives, friends, and acquaintances) have gone to the health facility for their pregnancy related care?” The response categories included: most, many, some, few and none. We recoded the categories as greater number (most or many), some and fewer number (few or none).

Using two questions each we created variables of the frequency of instrumental, emotional and informational support. These questions asked women during their last pregnancy how often there was someone to provide them with a particular support. Instrumental support questions included: “someone to help you with your daily chores” and 2) “someone to help you seek health care.” Emotional support items included: 1) “someone you could count on to listen to you when you had any problems, concerns, or fears” and 2) “someone who had gone through pregnancy and could understand what you were going through and be supportive of your experience.” Informational support included: 1) “someone who gave you good advice about a crisis or situation you were experiencing” and 2) “someone you could turn to when you needed suggestions and advice on how to deal with problems.” The response categories were: all of the time, most of the time, some of the time, a little of the time and none of the time. We recoded each of the categories as more of the time (all or most of the time) and less of the time (some, a little, or none of the time).

We included control variables known to be associated with use of health facility delivery including: maternal age, education, employment, household wealth, religion, marital status, ethnicity, parity, region, and decision-making autonomy [8]. We created the wealth variable based on a similar approach used in previous studies that examined baseline and midline data from the MNHR project [29, 33]. We selected three household characteristics: type of toilet, location of kitchen and type of fuel used. We coded as poorest households those that (1) use wood for fuel, (2) have non-improved toilet (definition from GDHS) and (3) a kitchen outside the house. We coded households with two out of three of these options as medium, and households with one or none of these options as richest. We derived the autonomy variable from the item: “who usually makes decisions about health care for you?” Response options were: respondent alone, husband/partner alone, respondent and husband/partner jointly, other network members. We recoded the options into high decision-making autonomy (respondent alone or respondent and husband/partner jointly), low decision-making autonomy (husband/partner alone), and others (other network members).

### Analysis

We conducted bivariate analyses of the association between each control variable and health facility birth, and also each network characteristic variable and facility birth, adjusting for the clustered survey design. Using separate logistic regression models we tested the association between each network characteristic and facility birth, adjusting for the control variables. We then ran separate logistic regression models to test two-way interactions, specifically whether network structure moderated the relationship between social support and/or social norms and health facility birth. We also tested whether the interaction between social support and social norms was associated with facility birth. We re-ran reduced models without interaction terms for models with insignificant interaction terms, and conducted post hoc analysis to probe the nature of the interaction of models with significant moderated effect [42]. All logistic regression analyses were two-tailed (*p* < 0.05) and adjusted for clustered survey design by including robust standard errors in SAS version 9.3 (SAS Institute, Cary NC).

## Results

Table 2 shows the descriptive characteristics of all women, and those who had homebirth or facility birth. Half of all respondents (50%) were between 25 and 34 years, and about 5% were younger than 19 years. The proportion of women with no formal education was significantly greater among respondents who had homebirth than those who had facility birth (69% vs. 32%, *p* < 0.01). A significantly greater proportion of women who had homebirth than those who had facility birth had unpaid work or were unemployed (81% vs. 53%, *p* < 0.01), were in the poorest household wealth category (66% vs. 41%, *p* < 0.01), were married or living with a partner (94% vs. 82%, *p* < 0.01), had six or more children (27% vs. 13%, *p* < 0.01), and indicated that their husband alone made decisions about their health care (67% vs. 59%, *p* < 0.01).

**Table 2:** Descriptive and network characteristics of women with most recent childbirth, and by place of birth

	Total sample (783)	Homebirth	Facility birth
Descriptive characteristics [*Percent*]	Percent (N)/ Mean (95% CI)	Percent (N)/ Mean (95% CI)	Percent (N)/ Mean (95% CI)
Age			
< 19 years 19–24 years 25–34 years 35–49 years	37 (4.7) 216 (27.6) 391 (49.9) 139 (17.8)	9 (2.8) 70 (21.6) 181 (55.9) 64 (19.8)	28 (6.1) 146 (31.8) 210 (45.8) 75 (16.3)***
Education			
None Primary Secondary	369 (47.3) 208 (26.6) 206 (26.3)	223 (68.8) 68 (21.0) 33 (10.2)	146 (31.8) 140 (30.5) 173 (37.7)****
Employment			
Paid Self employed Unpaid/ unemployed/other	36 (4.6) 240 (30.7) 507 (64.8)	7 (2.2) 55 (17.0) 262 (80.9)	29 (6.3) 185 (40.3) 245 (53.4)****
Household wealth			
Richest Medium Poorest	133 (17.0) 247 (31.6) 403 (51.5)	21 (6.5) 88 (27.2) 215 (66.4)	112 (24.4) 159 (34.6) 188 (41.0)****
Religion			
Christian Moslem None/traditional/Other	461 (58.9) 215 (27.5) 107 (13.7)	151 (46.6) 95 (29.3) 78 (24.1)	310 (67.5) 120 (26.1) 29 (6.3)****
Marital status			
Married/living together Not currently in union	681 (87.0) 102 (13.0)	304 (93.8) 20 (6.2)	377 (82.1) 82 (17.9)****
Ethnicity			
Akan Mole-Dadgbani Grum Other	337 (43.0) 193 (24.7) 106 (13.5) 147 (18.8)	72 (22.2) 89 (27.5) 69 (21.3) 94 (29.0)	265 (57.7) 104 (22.6) 37 (8.1) 53 (11.6)****
Parity			
1 2 3 4 5 6 +	185 (23.6) 143 (18.3) 139 (17.8) 92 (11.8) 76 (9.7) 148 (18.9)	47 (14.5) 46 (14.2) 59 (18.2) 47 (14.5) 37 (11.4) 88 (27.2)	138 (30.1) 97 (21.1) 80 (17.4) 45 (9.8) 39 (8.5) 60 (13.1)****
Region			
Central Northern	380 (48.5) 403 (51.5)	87 (26.9) 237 (73.1)	293 (63.8) 166 (36.2)****
Who usually makes decision about your health care			
Husband alone Respondent alone/ both partners Other	457 (67.1) 185 (27.2) 185 (5.7)	235 (77.3) 50 (16.5) 19 (6.2)	222 (58.9) 135 (35.8) 20 (5.3)****
**Network structure** [*Mean*]			
Network size	3.66 (3.36–3.96)	3.87 (3.52–4.22)	3.51 (3.16–3.85)
Number of network members that respondent interacted with	2.81 (2.56–3.07)	3.13 (2.82–3.44)	2.59 (2.34–2.85)
Number of network members that respondent lived near	1.59 (1.43–1.76)	1.69 (1.47–1.91)	1.52 (1.33–1.72)
**Social influence** [*Percent*]			
Close relatives approval of facility-based pregnancy and delivery care			
Lower approval Higher approval	153 (19.7) 625 (80.3)	89 (27.7) 232 (72.3)	64 (14.0) 393 (86.0)****
Close friends approval of facility-based pregnancy and delivery care			
Lower approval Higher approval	285 (36.6) 493 (63.4)	109 (33.8) 214 (66.2)	176 (38.7) 279 (61.3)
Number of women respondent know that have gone to a facility for pregnancy-related care			
Fewer number Some Greater number	77 (9.9) 204 (26.3) 495 (63.8)	55 (17.1) 102 (31.7) 165 (51.2)	22 (4.9) 102 (22.5) 330 (72.7)****
**Instrumental support** [*Percent*]			
There was someone to help with daily chores			
Less of the time More of the time	321 (41.0) 462 (59.0)	147 (45.4) 177 (54.6)	174 (37.9) 285 (62.1)*
There was someone to help seek health care			
Less of the time More of the time	264 (33.8) 518 (66.2)	131 (40.6) 192 (59.4)	133 (29.0) 326 (71.0)**
**Emotional support** [*Percent*]			
There was someone to listen, if respondent had any problems			
Less of the time More of the time	295 (37.7) 487 (62.3)	120 (37.0) 204 (63.0)	175 (38.2) 283 (61.8)
There was someone who could understand and support respondent through pregnancy			
Less of the time More of the time	298 (38.1) 485 (61.9)	131 (40.4) 193 (59.6)	167 (36.4) 292 (63.6)
**Informational support** [*Percent*]			
There was someone to give respondent advice			
Less of the time More of the time	277 (35.4) 506 (64.6)	122 (37.7) 202 (62.3)	155 (33.8) 304 (66.2)
There was someone respondent could turn to for suggestions on dealing with concerns			
Less of the time More of the time	299 (38.2) 484 (61.8)	131 (40.4) 193 (59.6)	168 (36.6) 291 (63.4)

Sample size is slightly smaller for some variables that had missing data. Significance tests compare homebirth with facility birth; *p ≤ 0.05; **p ≤ 0.01; ***p ≤ 0.001; ****p ≤ 0.0001

The descriptive table also included women’s network characteristics (Table 2). While not statistically significant, women who had homebirth had a higher mean network size (3.87 vs. 3.51), mean number of network members they interacted with (3.13 vs. 2.59), and mean number of network members they lived near (1.69 vs. 1.52), than the proportion of women who delivered in a facility.

In regards to social norms, a significantly greater proportion of respondents who had facility birth than the proportion that had homebirth perceived that their close relatives (86% vs. 72%, *p* < 0.01) had higher approval of facility-based pregnancy and delivery care. Additionally, 64% of all respondents perceived that a greater number of women they know have gone to a facility for pregnancy-related care. More women who had facility birth compared to those who had homebirth perceived that a greater number of women they know have gone to a facility for pregnancy related care (73% vs. 51%, *p* < 0.01).

With regard to instrumental support, compared to women who delivered at home, a significantly greater proportion of women who had facility birth perceived that more of the time there was someone to help them with daily chores (62% vs. 55%, *p* = 0.02); and to help them seek health care (71% vs. 59%, *p* < 0.01). There was no difference in emotional and instrumental support by place of delivery.

After controlling for women’s age, education, employment, household wealth, parity, marital status, religion, ethnicity, region and decision-making autonomy, women who perceived that their close relatives had a higher approval of facility-based pregnancy and delivery care were significantly more likely to have health facility birth (OR: 2.16, CI: 1.27–3.68) than those who perceived that their close relatives had a lower approval (Table 3, Model 4). Respondents who perceived that a greater number of women they know have gone to a facility for pregnancy-related care were significantly more likely to have a facility birth (OR: 2.20, CI: 1.21–4.00) than those who perceived that a fewer number of women they know have gone to a facility (Table 3, Model 6). Women who perceived that more of the time there was someone to help them seek health care were significantly more likely to have facility birth (OR: 1.60, CI: 1.10–2.34), compared with those who perceived that less of the time there was someone to help (Table 3, Model 8). Women who perceived that more of the time there was someone to give them advice were significantly more likely to have facility birth (OR: 1.66, CI: 1.08–2.54, [Table 3, Model 11]). The association between respondent’s perception that there was someone they could turn to for suggestions and facility birth was marginally significant at a *p*-value of 0.05 (OR: 1.51, CI: 0.99–2.28, [Table 3, Model 12]). Network structure and emotional support variables were not significantly associated with facility birth.

**Table 3:** Logistic regression odds ratios of association between network characteristics and health facility birth among women with most recent childbirth

	Model	Network characteristics	Health Facility birth
			AOR (95% CI)
Network structure	1	Network size	1.04 (0.97–1.12)
	2	Number of network members that respondent interacted with	0.98 (0.90–1.07)
	3	Number of network members that respondent lived near	1.07 (0.93–1.25)
Social influence	4	Close relatives approval of facility-based pregnancy and delivery care:	
		Lower approval Higher approval	1.0 2.16 (1.27–3.68)**
	5	Close friends approval of facility-based pregnancy and delivery care:	
		Lower approval Higher approval	1.0 1.30 (0.88–1.93)
	6	Number of women respondent know that have gone to a facility for pregnancy-related care:	
		Fewer number Some Greater number	1.0 1.85 (0.96–3.58) 2.20 (1.21–4.00)**
Instrumental support	7	There was someone to help with daily chores:	
		Less of the time More of the time	1.0 1.31 (0.95–1.81)
	8	There was someone to help seek health care:	
		Less of the time More of the time	1.0 1.60 (1.10–2.34)**
Emotional support	9	There was someone to listen, if respondent had any problems:	
		Less of the time More of the time	1.0 1.07 (0.71–1.63)
	10	There was someone who could understand and support respondent through pregnancy:	
		Less of the time More of the time	1.0 1.32 (0.93–1.87)
Informational support	11	There was someone to give respondent advice:	
		Less of the time More of the time	1.0 1.66 (1.08–2.54)*
	12	There was someone respondent could turn to for suggestions on dealing with concerns:	
		Less of the time More of the time	1.0 1.51 (0.99–2.28)*

Note: Regression model for each independent variable controlled for age, education, employment, household wealth, parity, marital status, religion ethnicity, region, and decision-making autonomy. *p ≤ 0.05; **p ≤ 0.01

**Fig. 2:**
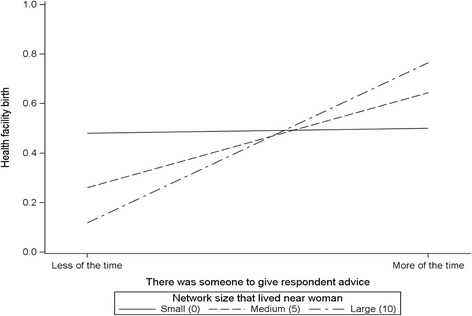
*Health facility birth by network structure and informational support* (number of network members that lived near respondent, and there was someone to give respondent advice)

Nearly all modelled interactions between each network structure and social support variable, and each network structure and social norm variable, were not significantly associated with facility delivery, adjusting for control variables. Also, most of the modeled interactions between each social norm and social support variable were not significantly associated with facility delivery. Table 4 presents two models that included the interaction between a network structure and social support variable, and a social norm and social support variable. Interaction model 1 included the social support variable “there was someone to give respondent advice” and network structure variable “number of network members that respondent lived near.” The interaction between these two variables had a marginally significant association with facility birth, *p*-value = 0.05 (OR: 1.37, CI: 1.00–1.87). This interaction, as depicted in Fig. 2, shows that the positive relationship between women’s perception that more of the time there was someone to give them advice and health facility birth was moderated by the number of network members that lived nearby. Among women who perceived that more of the time there was someone to give them advice, those who lived near large compared with small numbers of network members were more likely to have facility delivery.

**Table 4:** Logistic regression odds ratios of the association of interactions between network characteristics with health facility birth among women with most recent childbirth

	Facility delivery	
Network characteristics	Interaction model 1 AOR (95% CI)	Interaction model 2 AOR (95% CI)
Number of network members that respondent lived near	0.83 (0.65–1.05)	–
There was someone to give respondent advice:		
Less of the time More of the time	1.0 1.08 (0.59–1.98)	1.0 0.66 (0.23–1.92)
Number of women respondent know that have gone to a facility for pregnancy-related care:		
Fewer number Some Greater number	-- -- --	1 0.67 (0.28–1.61) 1.54 (0.59–4.01)
Number of network members that respondent lived near X There was someone to give respondent advice		
	1.37 (1.00–1.87) +	--
There was someone to give respondents advice X Number of women respondent know that have gone to a facility for pregnancy-related care:		
Fewer number Some Greater number	-- -- --	1 5.58 (1.64–19.02)** 0.65 (0.48–5.65)

Note: each regression model controlled for age, education, employment, household wealth, parity, marital status, religion ethnicity, region, and who usually make decision about your healthcare. Model 1 included interaction of network structure and social support, and Model 2 included the interaction of social support and social influence. *p ≤ 0.05; **p ≤ 0.01; + *p* = .052

**Fig. 3:**
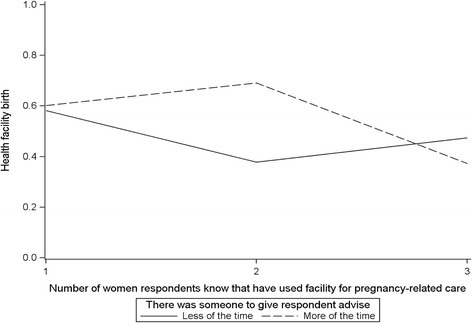
Health facility birth by informational support and descriptive norm *(number of women respondent know that have gone to a facility for pregnancy-related care, and there was someone to give respondents advice)*

Interaction model 2 (Table 4) includes the informational support variable “perception that there was someone to give respondent advice” and descriptive norm variable “number of women respondent know that have gone to a facility for pregnancy-related care.” The interaction between these two variables was significantly associated with facility birth (OR: 5.58, CR: 1.64–19.02, *p* value < 0.01). As depicted in Fig. 3, respondents’ perception that there was someone to give them advice modified the positive relationship between their perception that some women they know have gone to a facility for pregnancy-related care and their own use of facility birth. Among respondents who perceived that some women they know have gone to a facility for pregnancy-related care, those who perceived that more of the time there was someone to give them advice were more likely to have facility delivery.

## Discussion

Our findings indicate that social norms, instrumental support and informational support were positively associated with women’s use of health facility birth in Ghana. Also, social network structure (proximity) moderated the relationship between social support and facility delivery.

Similar to our examination of the interactive effect of network structure and function, Edmonds and colleagues (2012) previously assessed the interaction between women’s network structure and women’s perceptions of advice from network members to either deliver at home or at a health facility [25]. Drawing from the Network Episode Model, which posits that health decisions are made in the context of interpersonal interactions within one’s social network, Edmonds et al. (2012) suggested that interaction between network structure and perceptions of advice would help explain pregnant women’s decision to utilize health facility birth. These authors, however, did not find evidence of this interaction, which they interpreted as being due to their network structural variable failing to capture distinct network structural features of women in rural Bangladesh. In our analyses, we found that women who perceived that more of the time there was someone to give them advice and lived near large numbers of network members had a higher odds of facility birth.

Our finding of an interaction effect between informational support and network proximity suggests that network structure operates by modifying the relationship between social support and health-related behavior. Similar examples are found in the maternal health literature, which suggests that network structure interacts with network functions to influence maternal health [23, 43]. For example, in their study of women in rural Kenya, Kohler et al. (2001) found that network density moderated the relationship between social influence in the form of descriptive norms (number of contraceptive users in a woman’s social network) and contraceptive use [23]. The positive relationship between number of contraceptive users in a woman’s social network and women’s contraceptive use was stronger for women with a higher network density.

We found that network structure was not significantly associated with facility birth. But, contrary to our hypothesis, women who had homebirth had higher mean network size, frequency and proximity than those who had facility-birth. It is possible that women who gave birth at home interacted with a slightly higher number of women during their pregnancy and delivery experiences, and yet network members of women who had facility birth were more influential in the decision-making process and support provision to get women to a health facility. We also note that the mean network size was larger than the mean number of network members that lived close to women, but not much bigger than mean number of members that interacted with women. Women’s response to the question regarding their interactions with network members likely reflected face-to-face interaction, as the survey question did not emphasize other means of communication including mobile telephone use. Thus, during pregnancy women interacted with more people than just those who live in close proximity to them.

Few studies have explored the interaction between social support and social norms as an explanation of how network functions are associated with facility delivery [27, 32]. Ono and colleagues (2013) qualitatively examined the determinants of the effect of social support on both married and unmarried women’s use of facility delivery in Kenya [27]. They argued that married women lived in close-knit communities with their husbands’ family household and were likely influenced by the family’s normative belief in homebirths, whereas unmarried women were not subject to normative influences from a husband’s family to deliver at home, and so were less likely to experience homebirth. Our study builds on Ono and colleagues (2013) work by specifically examining the interactive effect of social support and social norms on facility birth. We found that among respondents who perceived that some of the women they know have gone to a facility for pregnancy-related care, those who perceived that more of the time there was someone to give them advice had a high probability of facility birth. This suggests that whereas women who perceived social norms favoring facility delivery were more likely to have facility birth, receiving advice from network members during pregnancy further strengthened this likelihood.

Consistent with previous research [25, 27], the measures of social support in our study revealed that women’s perception that there was someone to help them seek health care, and to give them advice during pregnancy was positively associated with facility delivery. Our finding is supported by previous work that examined the network functions of specific network members [44–46]. For instance, Moyer et al. (2014) qualitatively examined how social factors influence facility delivery in Ghana [47]. They found that women were dependent on their social networks including husband, mother-in-law and head of household for instrumental (economic or logistics) support to get them to a health facility for childbirth. Contrary to our hypothesis, we did not find a significant association between emotional support and facility delivery. Research on the relationship between emotional support and facility delivery is limited. Previous qualitative research in Bangladesh has found that women who received emotional support from their husbands during labor were more likely to have facility delivery [32]. Possibly, our measure of emotional support did not reflect the type of emotional support experienced by rural Ghanaian women.

In terms of social norms, we found that respondents’ perception that their close relatives approved of facility-based pregnancy and delivery care, and that women they know have gone to a facility for pregnancy-related care were positively associated with facility birth. This finding suggests that in addition to the growing evidence of a shift in social norms toward use of facility birth in Ghana [47, 48], the normative influence of women’s network members regarding facility delivery is directly associated with women’s use of health facility birth.

Limitations in our study are worth noting. Our analysis was based on cross-sectional data, which makes it impossible to infer causality between our key independent variables and outcome. Also, it is unknown whether participant’s perception of network approval of facility delivery may have been influenced by their actual experience of facility birth, rather than an earlier notion of network support for facility delivery. Longitudinal studies are needed to establish causality. Although clustering in our analysis results in less precision than, for example, a simple random sampling, this was the best approach to collect data from women in rural areas who were clustered in communities/ villages. Moreover, we controlled for the effects of clustering in analysis. We sampled women who had given birth 3 years prior to the survey administration, and this may have introduced an element of recall bias. We collected egocentric network data on women’s social network characteristics. As such we did not acquire information on the perspective of women’s own network members, which may have provided different insight into the network members’ role in facilitating facility delivery. While research has demonstrated that previous use of facility birth is an important predictor of health facility delivery [4, 8], we were unable to control for this variable, as the survey questions for this study were solely focused on women’s recent pregnancy and birth experiences.

## Conclusion

Network functions in women’s pregnancy experiences were associated with facility delivery. This demonstrates the importance of accounting for the roles of network members in supporting women’s pregnancy when designing maternal and child health interventions to promote use of facility-based pregnancy and delivery care. Maternal health interventions should be tailored to directly incorporate network members in strategies to increase uptake in women’s use of pregnancy related health services. Also, future research should examine which types of network members provide specific kinds of social support and are influential in facilitating women’s use of facility birth. In countries like Ghana, such work may have immediate impact by informing on-going national level interventions to improve health services delivery for pregnant women.
